# Biredox‐Ionic Anthraquinone‐Coupled Ethylviologen Composite Enables Reversible Multielectron Redox Chemistry for Li‐Organic Batteries

**DOI:** 10.1002/advs.202103632

**Published:** 2021-10-29

**Authors:** Zhongju Wang, Qianqian Fan, Wei Guo, Changchun Yang, Yongzhu Fu

**Affiliations:** ^1^ College of Chemistry Zhengzhou University Zhengzhou 450001 P. R. China

**Keywords:** anthraquinone, cathode, ethylviologen, lithium‐organic battery, redox activity

## Abstract

Organic compounds bearing redox‐active ionic pairs as electrode materials for high‐performance rechargeable batteries have gained growing attention owing to the properties of synthetic tunability, high theoretical capacity, and low solubility. Herein, an innovative biredox‐ionic composite, i.e., ethylviologen dianthraquinone‐2‐sulfonate (EV‐AQ_2_), affording multiple and reversible active sites as a cathode material in lithium‐organic batteries is reported. EV‐AQ_2_ exhibits a high initial capacity of 199.2 mAh g^−1^ at 0.1 C rate, which corresponds to the transfer of two electrons from one redox couple EV^2+^/EV^0^ and four electrons from two redox‐active AQ^−^ anions. It is notable that EV‐AQ_2_ shows remarkably improved cyclability compared to the precursors. The capacity retention is 89% and the Coulombic efficiency approaches 100% over 120 cycles at 0.5 C rate. The results offer evidence that AQ^−^ into the EV^2+^ scaffold with multiple redox sites are promising in developing high‐energy‐density organic rechargeable batteries.

## Graphical abstract

A biredox ionic ethylviologen dianthraquinone‐2‐sulfonate (EV‐AQ_2_) consisting of both redox cations and anions is synthesized. EV‐AQ_2_ can undergo reversible multielectron redox reactions to improve their stability and theoretical capacity, which corresponds to two electrons from EV^2+^ cations and four electrons from AQ^−^ anions. This study illustrates an effective strategy to design new ionic organic compounds for Li‐organic batteries.

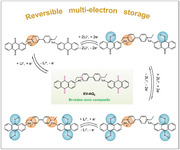

## 1 Introduction

Electrochemical energy storage technologies have played an important role in the storage of renewable solar and wind power.^[^ ^1^, ^2^ ^]^ In this respect, redox‐active organic electrode materials have gradually attracted more attention in rechargeable batteries for energy storage, owning to their adjustable structure, high theoretical capacity, ubiquity, and low cost.^[^ ^3^, ^4^ ^]^ To date, several organic electrode materials have been proposed, mainly including conductive polymers,^[^ ^5^, ^6^ ^]^ organic radical compounds,^[^ ^7^, ^8^ ^]^ carbonyl compounds,^[^ ^9^, ^10^, ^11^, ^12^, ^13^ ^]^ and organosulfide compounds.^[^ ^14^, ^15^, ^16^ ^]^ Organic materials can undergo unique electrochemical conversion processes to satisfy correlative requirements in various metal‐ion (such as Li^+^, Na^+^, K^+^, and Mg^2+^) batteries/metal batteries, endowing distinctive electrochemical performance in terms of voltage, capacity, cycling stability, and rate performance.^[^ ^17^, ^18^, ^19^, ^20^ ^]^ However, many of these organic materials in batteries with aprotic organic electrolyte often face several challenges, such as dissolution in electrolytes, drain of electrochemical activity, and slow kinetics, which are primarily related to their weak stability during cycling in batteries.^[^ ^21^ ^]^ Therefore, avoiding the solubility issue in common organic electrolyte and improving the electrochemical performance require the optimization of organic molecular structure. Among various strategies, polymerization and covalent grafting have been extensively attempted to restrict solubility and enhance the cycling stability of active organic molecules.^[^ ^22^ ^]^ However, this method often needs complex synthetic routes and undergoes several reaction steps to introduce electrochemically inactive substituents, resulting in low density of redox active groups attached to polymer backbones.^[^ ^23^ ^]^ Consequently, other effective strategies are urgently needed to overcome these inevitable limitations, i.e., solubility and low stability of organic active materials.

As a unique category of salification to suppress the dissolution issue, organic salts forming with carboxylates, quinone salts, imide salts, and sulfonates display high polarity, which is beneficial for their cycling stability.^[^ ^24^, ^25^, ^26^, ^27^, ^28^, ^29^ ^]^ For example, anthraquinone‐1,5‐disulfonic acid sodium salt with the strong electron withdrawing group shows low solubility and stable electrochemical performance.^[^ ^30^ ^]^ However, nonredox‐active inorganic cations (e.g., Na^+^) are incorporated in accompany with redox‐active organic anions, which inevitably results in the reduction of specific capacity. More recently, viologens and their derivatives as electron acceptor cations, which can undergo reversible redox reactions, have been explored for electrochemical energy storage.^[^ ^31^, ^32^, ^33^ ^]^ In light of this, the approaches prompt us to construct ionic organic compounds consisting of both redox cations and anions which can undergo reversible multielectron redox reactions to improve their stability and theoretical capacity.

**Scheme 1:**
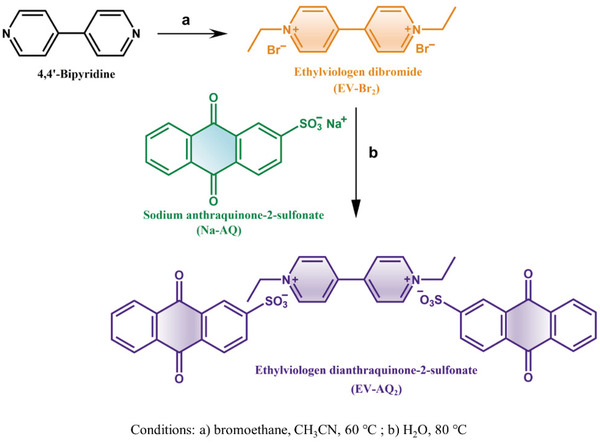
Synthetic route of ethylviologen dianthraqui‐none‐2‐sulfonate (EV‐AQ_2_) composite.

Herein, we report a biredox ionic ethylviologen dianthraquinone‐2‐sulfonate (EV‐AQ_2_), which is made of anthraquinone‐2‐sulfonate (AQ^−^) coupled with ethylviologen (EV^2+^) moieties. As a cathode material for lithium batteries, it could achieve multielectron storage. The innovative introduction of the unique *π*‐framework AQ^−^ to the EV^2+^ structure makes it possible to enhance interactions between the molecules.^[^ ^34^ ^]^ The synthetic route of EV‐AQ_2_ is shown in **Scheme** **1**, with a detailed information depicted in the experimental section (Supporting Information). When introducing anionic AQ^−^ to couple with cationic EV^2+^ instead of the halogen ion, e.g., Br^−^or I^−^, through a facile anion‐exchanged method, the theoretical specific capacity of EV‐AQ_2_ is 203.8 mAh g^−1^, corresponding to the transfer of four‐electrons from two anions AQ^−^ and two‐electrons from one thread‐like cation EV^2+^. Based on the molecular design of this new material, EV‐AQ_2_ exhibits reversible performance and stable cycle life in rechargeable lithium batteries.

## 2 Results and Discussion

**Figure 1:**
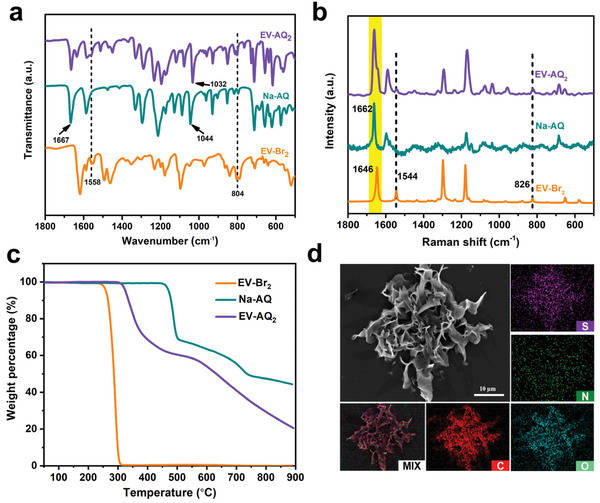
a) FTIR spectra, b) Raman spectra, and c) TGA curves of EV‐Br_2_, Na‐AQ, and EV‐AQ_2_. d) SEM and EDS images of EV‐AQ_2_.

The structure, purity, and thermal stability of the as‐obtained compound are confirmed by ^1^H nuclear magnetic resonance (^1^H NMR), Fourier transform infrared (FTIR) spectroscopy, Raman spectroscopy, and X‐ray diffraction (XRD) as well as thermogravimetric analysis (TGA). As shown in Figures S1–S3 in the Supporting Information, the ^1^H NMR spectra of ethylviologen dibromide (EV‐Br_2_), ethylviologen diiodide (EV‐I_2_), and EV‐AQ_2_ are collected in D_2_O, confirming the presence of EV^2+^ in EV‐Br_2_ and EV‐I_2_, and EV‐AQ_2_ coupled with AQ^−^ adequately. Meanwhile, the FTIR spectra of EV‐Br_2_, sodium anthraquinone‐2‐sulfonate (Na‐AQ), and EV‐AQ_2_ are depicted in **Figure** **1**a. The peaks at 804 and 1558 cm^−1^ in EV‐Br_2_ and EV‐AQ_2_ attributed to the in‐plane bending frequency and the C—H stretching vibration of 4‐substituted pyridine indicate the existence of EV^2+^.^[^ ^35^ ^]^ The peak at 1667 cm^−1^ assigned to the stretching vibration of C═O bond is shown in Na‐AQ and EV‐AQ_2_.^[^ ^36^ ^]^ Moreover, the peak attributed to the stretching vibration of the sulfonate substituent groups is also shown in EV‐AQ_2_ at 1032 cm^−1^, as observed in Na‐AQ at 1044 cm^−1^.^[^ ^37^ ^]^ Additionally, the full FTIR spectrum of EV‐AQ_2_ is also provided (Figure S4, Supporting Information) to verify the absence of remaining NaBr salt in EV‐AQ_2_. No characteristic peak signal (2925 cm^–1^) of NaBr appears, indicating that the obtained EV‐AQ_2_ is pure. The Raman spectrum (Figure 1b) of the as‐prepared EV‐AQ_2_ at 1662 cm^−1^ corresponds to the stretching vibration of C═O bond from the anion AQ^−^.^[^ ^38^ ^]^ The peaks at 1646 and 1544 cm^−1^ are attributed to the C−C ring vibrations and vibrational modes of ethyl groups in EV^2+^, respectively.^[^ ^39^ ^]^ These results indicate the distinct coupling between the EV^2+^ and AQ^−^ species. From the TGA curves (Figure 1c), the thermal decomposition temperatures of EV‐Br_2_, Na‐AQ, and EV‐AQ_2_ samples are 240, 460, and 304 °C, respectively. Obviously, the thermal stability of EV‐AQ_2_ is better than that of EV‐Br_2_, suggesting EV^2+^ cations bearing AQ^−^ anions are beneficial for the thermal stability. The XRD pattern of EV‐AQ_2_ exhibits multiple crystalline structure which are different from those of EV‐Br_2_ and Na‐AQ (Figure S5, Supporting Information).

Scanning electron microscopy (SEM) and energy‐dispersive X–ray spectroscopy (EDS) were carried out to obtain the detailed morphology and elemental composition. As shown in Figure 1d, EV‐AQ_2_ powder exhibits flower‐like morphology, which is beneficial for electrolyte permeation and easily intertwined with carbon nanotubes (CNTs). Four elements of C, O, S, and N are uniformly distributed in EV‐AQ_2_ from the corresponding EDS images. To improve the conductivity of EV‐AQ_2_ in lithium batteries, we construct the free‐standing, flexible, and binder‐free EV‐AQ_2_/CNTs cathode via a facile dissolution‐recrystallization method.^[^ ^40^ ^]^ The detailed preparation processes are shown in the experimental section and the cell configuration is shown in Figure S6 in the Supporting Information. CNTs are selected due to their excellent electrical conductivity and appropriate porosity for increasing the electrode/electrolyte contact.^[^ ^41^ ^]^ In addition, they can physically confine with EV‐AQ_2_. As shown in the SEM images in Figure S7 in the Supporting Information, the EV‐Br_2_, Na‐AQ, and EV‐AQ_2_ are dispersed well in the carbon nanotube networks. Furthermore, these ionic crystals are insoluble in the G4 solvent through the solubility tests (Figure S8, Supporting Information).

**Figure 2:**
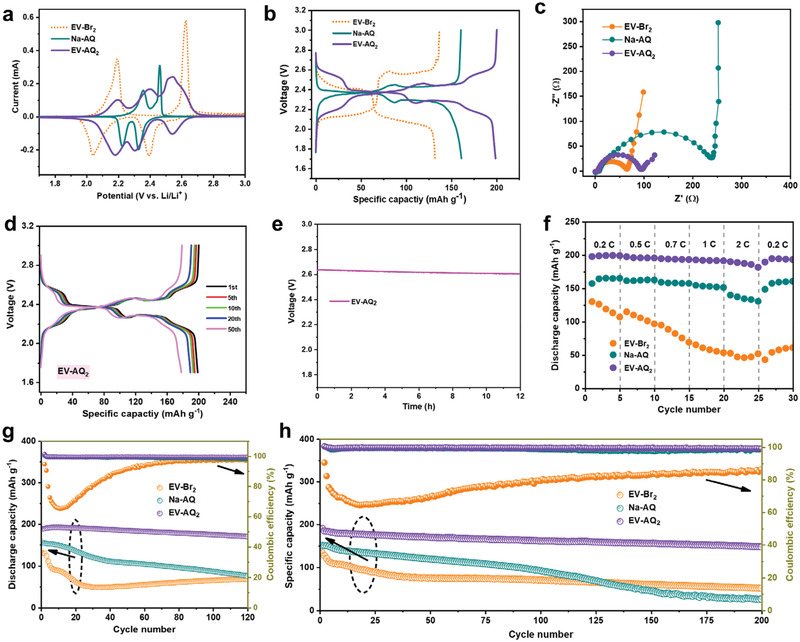
a) Cyclic voltammogram (CV) curves of different cathodes in a voltage window of 1.7–3.0 V at a scan rate of 0.1 mV s^−1^. b) Voltage profiles of different cathodes with same mass loading in the first cycle at 0.1 C. c) Nyquist plots of the EV‐Br_2_, Na‐AQ, and EV‐AQ_2_ electrode at open‐circuit voltage (OCV) of the cells. d) Galvanostatic charge/discharge profiles of EV‐AQ_2_ cathode during different cycles at 0.1 C (1 C = 203.8 mA g^−1^). e) Self‐discharge curve of EV‐AQ_2_ in 2 m LiTFSI/G4 electrolyte. The open circuit voltage kept stable after 12 h. f) Rate capability of different cathodes at different current densities. g) Cycling performance and Coulombic efficiency of different cathodes at 0.5 C. h) Cycling performance of different cathodes at 1 C. The C rate of each material is calculated based on its theoretical specific capacity, which is 143.3, 172.8, and 203.8 mAh g^−1^ for EV‐Br_2_, Na‐AQ, and EV‐AQ_2_, respectively.

To evaluate the electrochemical performances of EV‐Br_2_, Na‐AQ, and EV‐AQ_2_ as cathode‐materials for rechargeable lithium batteries, coin cells were assembled with 2 m lithium bis(trifluoro‐methanesulfonyl)imide (LiTFSI) in G4 as electrolyte. **Figure** **2**a displays the CV curves of three ionic molecules in the first cycle at 0.1 mV s^−1^ between 1.7–3.0 V versus Li/Li^+^. When using EV‐Br_2_ cathode, two single‐electron‐transfer steps are identified with corresponding reduction peaks at 2.40 V and 2.04 V.^[^ ^42^, ^43^ ^]^ However, by observing the details (Figure S9a, Supporting Information), the reduction peak located at 2.04 V gradually shifts to 2.12 V, while the reduction peak located at 2.40 V gradually shifts to 2.53 V, suggesting the electrochemical changes occur during the redox process of EV‐Br_2_ cathode. These electrochemical changes are believed to be related to the ionic exchange between LiTFSI and EV‐Br_2_ during cycling, forming a new pair of ionic compounds EV(TFSI)_2_ and LiBr. However, both of them are soluble in electrolyte, resulting in the continuous attenuation of capacity, and the decline of Coulombic efficiency. To confirm the ionic exchange of LiTFSI with EV‐Br_2_, both EV(TFSI)_2_ cathode and LiBr cathode are evaluated in the CV measurement for comparison. The CV curves of EV‐Br_2_/EV(TFSI)_2_ and LiBr/EV‐Br_2_ are shown in Figure S9b in the Supporting Information. For Br^–^, it cannot be oxidized during the voltage range of 1.8–3.0 V.^[^ ^44^ ^]^ For EV^2+^, the reduction‐peaks of EV‐Br_2_ after 50 cycles are basically located at the same positions of EV(TFSI)_2_, i.e., 2.53 and 2.12 V, which are different from their initial positions. It reveals that the ionic rearrangement of EV‐Br_2_ leads to gradual formation of EV(TFSI)_2_. Based on the peak‐splitting situation, the ion exchange might not be complete, EV‐Br_2_ and EV(TFSI)_2_ are both present in the cell. Moreover, EV(TFSI)_2_ accompanied with LiBr are soluble in ether electrolyte, which is not beneficial for the cycling stability. In contrast, the redox properties of the as‐obtained EV‐AQ_2_ with redox cation/anion pairing clearly point to stable multielectron redox reactions during cycling as we expected (Figure 2a). Remarkably, the CV curves of EV‐AQ_2_ cathode are almost overlapped at 2.54 V/2.53 V, 2.40 V/2.31 V, and 2.20 V/2.18 V after 50 cycles without apparent peak shifts, demonstrating high reversibility and outstanding stability of EV‐AQ_2_ due to its insolubility (Figure S10a, Supporting Information). Meanwhile, the CV curve of Na‐AQ has two pairs of redox peaks at 2.46 V/2.33 V and 2.36 V/2.23 V, which remain unchanged upon cycling without showing the ionic rearrangement (Figure S10b, Supporting Information). So, the combination of AQ^−^ with cations (EV^2+^ and Na^+^) is more stable than TFSI^−^ and not easily exchanged. These CV results also distinctly confirm multiple redox reactions and electrochemical reversibility of EV‐AQ_2_ as cathode for Li‐batteries.

The galvanostatic charge/discharge curves of different cathode in the first cycle are shown in Figure 2b. EV‐Br_2_ cathode displays two plateaus at 2.41 and 2.07 V. However, it shows the changes of the discharge plateaus from 2.41 to 2.54 and 2.07 to 2.13 V within 50 cycles, coincident with its CV curves (Figure S11, Supporting Information). The ionic rearrangement results in rapid fading of discharge capacity in the subsequent cycles. As a validation, when I^−^ instead of Br^−^ is introduced, the charge/discharge curves of EV‐I_2_ cathode are similar to those of EV‐Br_2_ cathode (Figure S12, Supporting Information), showing that analogous electrochemical reaction also occurs in EV‐I_2_ cathode in a voltage window of 1.7–3.0 V. After anionic exchange with TFSI^−^, the dissolution of new ionic compounds in the electrolyte results in the attenuation of capacity. With respect to Na‐AQ cathode, a discharge capacity of 161.4 mAh g^−1^ is delivered at a current rate of 0.1 C in the first cycle, which exhibits two discharge plateaus at 2.38 and 2.29 V corresponding to the two‐step reduction reactions of the carbonyl group to the enolates. In terms of multiple cycles (Figure S13, Supporting Information), Na‐AQ cathode displays no obvious change of two discharge plateaus due to the stability of its molecular structure. Significantly, the EV‐AQ_2_ cathode displays four discharge plateaus at 2.56, 2.38, 2.29, and 2.18 V, confirming multiple redox reactions. Moreover, the EV‐AQ_2_ can deliver a high specific capacity of 199.2 mAh g^−1^ in the first discharge process, corresponding to 97.7% of its theoretical value, which is higher than that (93.4%) of Na‐AQ. The different reaction kinetics for Na‐AQ and EV‐AQ_2_ cathodes can account for the different specific capacity values. By comparing the electrochemical impedance of the EV‐Br_2_, Na‐AQ, and EV‐AQ_2_ cathodes (Figure 2c), it can be seen that the charge transfer resistance (≈85 Ω) of EV‐AQ_2_ cathode is much lower than that (≈243 Ω) of Na‐AQ cathode. Additionally, the charge transfer resistance is closely related to the electrode reaction kinetics. Therefore, it can be inferred that the reaction kinetics of EV‐AQ_2_ is superior to that of Na‐AQ, which is manifested in that the EV‐AQ_2_ cathode can release higher initial capacity than that of Na‐AQ. The in situ electrochemical impedance of EV‐AQ_2_ cathode during cycling is also displayed in Figure S14 in the Supporting Information, the charge transfer impedance of EV‐AQ_2_ cathode gradually decreases during cycles, confirming the kinetic promotion of EV‐AQ_2_ cathode. The redox reaction kinetics of EV‐AQ_2_ cathode was further evaluated by CV test of EV‐AQ_2_ electrode at different scan rates (Figure S15a, Supporting Information). The voltammetric responses (currents vs scan rates) indicate that EV‐AQ_2_ cathode shows fast kinetic and pseudocapacitive behavior (*b* > 0.5) without the diffusion control (Figure S15b, Supporting Information). Importantly, the EV‐AQ_2_ cathode shows relatively stable charge/discharge voltage profiles and specific capacities during different cycles (Figure 2d), demonstrating a high redox reversibility of EV‐AQ_2_ in lithium batteries without the structural changes. Furthermore, the EV‐AQ_2_ cathode exhibits relatively low self‐discharge rate in 12 h, which is associated with the structural stability of EV‐AQ_2_ in the electrolyte (Figure 2e).

The rate performances of EV‐Br_2_, Na‐AQ, and EV‐AQ_2_ cathodes are evaluated at various C‐rates in Figure 2f. At current densities of 0.2 C, 0.5 C, 0.7 C, 1 C and 2 C, EV‐AQ_2_ cathode could maintain average discharge capacities of 199, 196, 194, 192, and 184 mAh g^−1^, respectively. When the rate decreases back to 0.2 C, the capacity can return to 194 mAh g^−1^, further suggesting the EV‐AQ_2_ has excellent rate performance and redox reversibility. Similarly, the Na‐AQ cathode delivers a good electrochemical activity due to the limited dissolution during cycling. Different from the coupling of EV^2+^ cations and AQ^−^ anions in EV‐AQ_2_, EV‐Br_2_ cathode shows dramatically decreased capacity during the cycling at diverse current densities due to the structural instability of EV^2+^ cations and Br^−^ anions during electrochemical processes.

The cycling performances and Coulombic efficiencies of EV‐Br_2_, Na‐AQ, and EV‐AQ_2_ cells at 0.5 C are presented in Figure 2g. EV‐Br_2_ cathode delivers the initial discharge of 130.2 mAh g^−1^ and Coulombic efficiency of 94.7%, thereafter suffering from a rapid discharge capacity decay, which is due to the severe loss and propagation of soluble redox active material, i.e., EV(TFSI)_2_, upon cycling. Although Na‐AQ cathode affords a stable cycle performance in the first ten cycles, it also undergoes a gradual capacity decay in the subsequent cycles, retaining 48% of its initial capacity (155.1 mAh g^−1^) after 120 cycles. This phenomenon may be caused by the inadequate anchoring of the active material to the surfaces of conductive CNTs during charge and discharge processes.^[^ ^45^ ^]^ On the contrary, EV‐AQ_2_ cathode delivers a high initial discharge capability of 188.2 mAh g^−1^. The capacity of EV‐AQ_2_ cathode slightly increases during the initial ten cycles, which is mainly contributed by the electrochemical activation process. Importantly, after 120 cycles at 0.5 C, EV‐AQ_2_ cathode can maintain a high capacity retention of 89% with the Coulombic efficiencies close to 100%. Notably, EV‐AQ_2_ cathode displays the improved electrochemical cycling stability at 1 C (Figure 2h). The specific capacity of 184.8 mAh g^−1^ in the first cycle decreases to 148 mAh g^−1^ after 200 cycles cathode, yielding a high capacity retention of 80%, which is much higher than those of EV‐Br_2_ and Na‐AQ cathodes. To demonstrate the capability of EV‐AQ_2_ in practical applications, we further increased the EV‐AQ_2_ mass loading to 3.0 mg cm^–2^ (Figure S16, Supporting Information), whereupon, a high capacity of 154.7 mAh g^−1^ at 0.5 C was obtained. The admirable performances of EV‐AQ_2_ cathode are ascribed to the enhanced structure stability of ionic EV‐AQ_2_ composite consisting of aromatic EV^2+^ and AQ^−^ units. Compared with Na‐AQ, the unique aromatic structure of EV^2+^ and AQ^−^ in EV‐AQ_2_ allows them to be strongly tethered on the surface of CNTs network via *π*−*π* interactions. This suppresses the drastic diffusion of the active materials into liquid electrolyte and promotes conductivity and structural stability of the electrode.

In order to verify the pre‐synthesized EV‐AQ_2_ and EV‐AQ_2_ obtained by spontaneous ion‐exchange during the cycles, a series of tests were carried out. First, the electrochemical properties of the mixed Na‐AQ/EV‐Br_2_ cathode were investigated in view of the evolution of the two active ions in the electrochemical reaction. The CV curves and galvanostatic charge/discharge curves of the mixed‐cathode within the voltage range of 1.7–3.0 V are displayed in Figure S17 in the Supporting Information. In the first cathodic scan (Figure S17a, Supporting Information), four distinct peaks appear corresponding to two single‐electron‐transfer steps from EV^2+^ in EV‐Br_2_ and two single‐electron‐transfer steps from AQ^−^ in Na‐AQ. Intriguingly, the reduction peaks of EV‐Br_2_ in the mixed‐cathode seem to shift to 2.54 and 2.13 V in subsequent scanning, implying intermolecular rearrangement between EV‐Br_2_ and Na‐AQ, further resulting in the variational redox processes. Furthermore, according to galvanostatic charge/discharge curves of the mixed‐cathode (Figure S17b, Supporting Information), there is an apparent change in discharge voltage plateaus located at 2.55, 2.38, 2.29, and 2.18 V after the first cycle, which is similar to that of pure EV‐AQ_2_ cathode. However, the mixed‐cathode at 0.2 C shows obvious discharge capacity decay from 118 to 61 mAh g^−1^ and the Coulombic efficiency drops from 113% to 96% after 150 cycles (Figure S18, Supporting Information). The inferior performances could be caused by incomplete structural rearrangement and heterogeneous distribution of EV‐AQ_2_ in the mixed‐cathode. As a result, the molecule combining strategy endows ionic EV‐AQ_2_ composite affording high theoretical capacity, good rate performance, and improved cycling stability in rechargeable lithium batteries.

**Figure 3:**
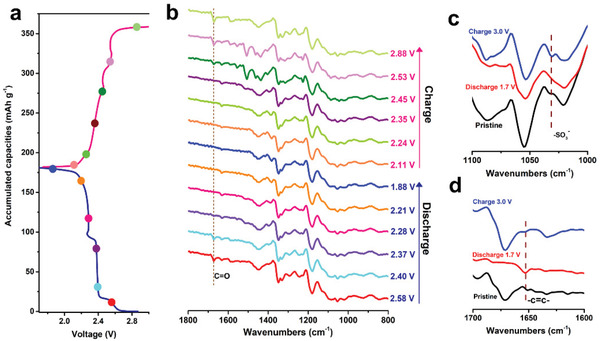
a) Galvanostatic charge/discharge profiles of EV‐AQ_2_ cathode at 0.1 C with marked points at different discharge and charge states in the in situ FTIR tests. b) In situ FTIR spectra of EV‐AQ_2_ cathode taken at different states as marked in a). FTIR spectra in selected wavenumbers range of c) 1100–1000 cm^−1^ and d) 1700–1600 cm^−1^ of pristine EV‐AQ_2_, discharged EV‐AQ_2_ at 1.7 V, and charged EV‐AQ_2_ at 3.0 V from in situ FTIR results.

To demonstrate the reaction mechanism of EV‐Br_2_, Na‐AQ, and EV‐AQ_2_ cathodes, we performed in situ FTIR spectroscopy to identify vibration absorption changes associated with multiple electron transfer and lithium storage during battery operations. As illustrated in Figure S19 in the Supporting Information, after discharge to 1.80 V, the strong enhancement peak of C═C stretching mode at 1652 cm^−1^ reflects EV^0^ emergence from EV^2+^ in EV‐Br_2_ cathode.^[^ ^46^ ^]^ Upon charging, the intensity of the peak at 1652 cm^−1^ becomes gradually weak, thus demonstrating the electrochemical reversibility of EV^2+^ cations. Meanwhile, the vibration of the C═O bonds at 1667 cm^−1^ in Na‐AQ cathode disappears and recovers completely when undergoing recycling, indicating the good recovery of carbonyl groups of AQ^−^ anions in the lithiation reactions (Figure S20, Supporting Information).^[^ ^47^ ^]^ As shown in **Figure** **3**a,b, EV‐AQ_2_ cathode exhibits the characteristic peaks at 1667 cm^−1^ in the initial stage, which is assigned to the carbonyl groups in EV‐AQ_2_. As expected, the in situ FTIR spectra exhibit clearly that the characteristic peak gradually disappears upon discharging, indicating the carbonyl groups as anionic active redox centers in EV‐AQ_2_ are successively lithiated like Na‐AQ. Upon charging to 2.88 V, the carbonyl groups signal appears in the original position, demonstrating the reversible characteristic of AQ^−^. The result shows that the EV‐AQ_2_ composite does not change the conversion mechanism of carbonyl groups. Furthermore, we focus on the characteristic peaks at 1652 and 1032 cm^−1^ in EV‐AQ_2_, which offers important information for the stretching vibration of C═C groups and the sulfonate substituent groups, respectively. The return of the sulfonate substituent groups at 1032 cm^−1^ at the end of charge reflects the reversible coupling of EV^2+^ and AQ^−^ (Figure 3c).^[^ ^48^ ^]^ Moreover, the characteristic peak at 1652 cm^−1^ in EV‐AQ_2_ appears and disappears after discharge to 1.7 V and recharge to 3.0 V, respectively, indicating the active center of EV^2+^ are used completely (Figure 3d). Hence, the in situ FTIR spectra verify the fact that EV‐AQ_2_ coupling with EV^2+^ and AQ^−^ is able to achieve the structural reversibility and outstanding electrochemical performances.

To further verify the structural reversibility of EV‐AQ_2_ after cycles, multiple characterizations were conducted. In the UV–vis investigation (Figure S21, Supporting Information), the G4 solution with the recharged EV‐Br_2_ electrode exhibits dark blue due to the dissolution of EV^+•^, by contrast, the G4 solution with the recharged EV‐AQ_2_ cathode is much lighter. In addition, the UV absorption signal of EV^+•^ in the recharged EV‐AQ_2_ electrode solution is not obvious, which indicates the structure of EV‐AQ_2_ is much more stable than EV‐Br_2_ during discharge, meaning almost no structural dissociation, so the structure of EV‐AQ_2_ is reversible when recharged. The dissolution and diffusion in EV‐Br_2_ and EV‐AQ_2_ cathodes can be further probed by the cycled Li foils extracted after several cycles (Figure S22, Supporting Information). It can be seen that the Li anode paired with EV‐Br_2_ cathode has been severely corroded, which could be EV‐Br_2_ discharged products (EV^+•^) migrated to the Li anode. In contrast, the surface of the Li anode paired with EV‐AQ_2_ cathode is smooth, implying the strong structural stability of EV‐AQ_2_. In addition, the morphology of the recharged EV‐AQ_2_ cathode has not changed much compared with the pristine EV‐AQ_2_ cathode after several cycles (Figure S23, Supporting Information). XRD patterns of the discharged and recharged EV‐AQ_2_ cathode (Figure S24, Supporting Information) are also displayed. However, since most of EV‐AQ_2_ are embedded in the carbon nanotube network, resulting in poor crystallization peaks of the pristine EV‐AQ_2_, discharged and recharged cathodes after several cycles.

**Figure 4:**
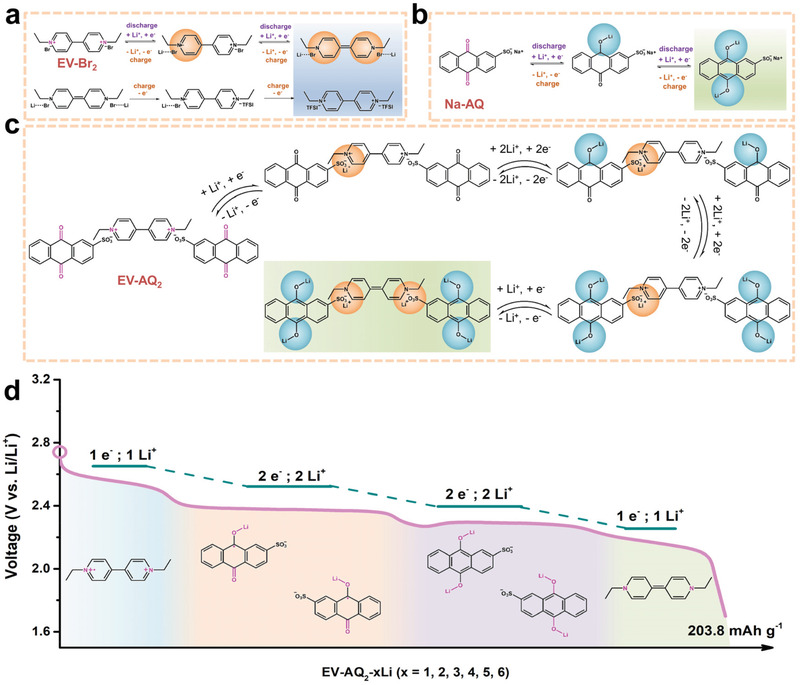
a–c) The proposed electrochemical redox reactions of EV‐Br_2_ cathode, Na‐AQ cathode, and EV‐AQ_2_ cathode in Li‐battery during discharging/charging process. Different colors are used to distinguish redox‐active EV^2+^ cations and redox‐active AQ^−^ anions. d) Schematic diagram of multielectron discharging process of EV‐AQ_2_ cathode.

According to the above electrochemical performances and in situ FTIR spectra of EV‐Br_2_, Na‐AQ, and EV‐AQ_2_ cathode, we briefly propose electrochemical mechanisms of the three cathodes during lithium storage. As shown in **Figure** **4**a, for EV‐Br_2_ cathode, the upper process represents the bonding of Li‐Br and the formation of neutral EV^0^ during the discharge process. In the charge process, partial TFSI^–^ anions may couple with EV^+•^ or EV^2+^ from the oxidation of EV^0^. Then, the EV‐Br_2_/Li cell suffers from a rapid capacity decay in the electrochemical reaction because of the generated EV(TFSI)_2_ and detached LiBr, both of which can dissolve in the electrolyte. As for Na‐AQ cathode in Figure 4b, two electron redox processes are fully reversible. Considering one EV^2+^ and two AQ^−^ electroactive centers in the EV‐AQ_2_ molecule, we propose that a total six electrons storage occurs upon discharge, including the transfer of two electrons from the reduction of one dication EV^2+^ to neutral EV^0^ in viologen and four electrons from the reduction of two pairs of carbonyl groups in anthraquinone‐2‐sulfonate (Figure 4c). More importantly, the evolution of EV‐AQ_2_ cathode demonstrates the presence of two AQ^−^ anions could lead to potential aromatic‐interactions between the cations and anions during discharging/charging, further implying complete conversion and structural stability of EV‐AQ_2_. Therefore, each lithiation process of EV‐AQ_2_ corresponding to discharged voltage plateaus at 2.56, 2.38, 2.29, and 2.18 V, respectively, are illustrated in Figure 4d. The first discharge plateau at 2.56 V should correspond to the dication EV^2+^ to radical cations EV^+•^ along with a Li^+^ ion pairing with —SO_3_^−^ in AQ^−^, the second discharge plateau at 2.38 V corresponds to the reduction of one pair of carbonyl group in two anion pendants of AQ^−^. Then, the third discharge profile is observed at 2.29 V, associated with the reduction of other one pair of carbonyl group. Finally, the neutral EV^0^ is formed by the conversion of the radical cations EV^+•^ at 2.18 V. The voltage plateaus demonstrate that EV‐AQ_2_ achieve a total six electrons, thus yielding a theoretical specific capacity of 203.8 mAh g^−1^.

## 3 Conclusion

In summary, we demonstrate a unique reversible six electrons storage towards Li batteries employing EV‐AQ_2_ containing two kinds of redox‐active groups EV^2+^ and AQ^−^ in the structure. The impressive high cycling stability and excellent rate capability are demonstrated for the as‐obtained EV‐AQ_2_ system in 2 m LiTFSI/G4 between 1.7 and 3.0 V compared to the EV‐Br_2_ and Na‐AQ materials. EV‐AQ_2_ can store total six electrons per formula, thereby exhibiting a high initial capacity of 199.2 mAh g^–1^ at 0.1 C rate. Notably, over 200 cycles at a high current rate of 1 C, a reversible capacity of 148 mAh g^−1^ with a capacity retention of 81% is observed. Through in situ FTIR, the reversible charge/discharge mechanism has been demonstrated. Therefore, this work shows that EV‐AQ_2_ endows a biredox couple as a promising cathode for promoting the development of multielectron storage in high performance lithium batteries.

## 4 Experimental Section

### Materials

4,4’‐Bipyridine (C_10_H_8_N_2_, 98%, Adamass), bromoethane (C_2_H_5_Br, 99%, Adamass), iodoethane (C_2_H_5_I, 99%, Adamass), sodium anthraquinone‐2‐sulfonate (C_14_H_7_NaO_5_S, 98%, Adamass), tetraehtylene glycol dimethyl ether (G4, 99%, Canrd), lithium bis(trifluoromethanesulfonimide) (LiTFSI, 99.95%, Sigma‐Aldrich) were used.

### Synthesis of EV‐Br_2_

The EV‐Br_2_ sample was synthesized according to the literature.^[^ ^49^ ^]^ In a typical procedure, 4,4’‐bipyridine (1.0 g, 6.4 mmol) was added dropwise to bromoethane (2.1 g, 19.2 mmol) in 20 mL of acetonitrile under argon atmosphere. The mixture was stirred at room temperature for 2 h and then heated at 60 °C for 12 h. After cooling, a yellow precipitate was obtained and separated by filtration. The crude product was washed three times with anhydrous acetonitrile and then washed three times with anhydrous ether. Finally, the resultant yellow pure product was dried under vacuum at 60 °C for 24 h (remarked as EV‐Br_2_). The ethylviologen diiodide (EV‐I_2_) sample was obtained by the same procedure above, using iodoethane instead of bromoethane. In addition, ethylviologen dibromide (EV(TFSI)_2_) was synthesized by dropping LiTFSI aqueous solution into as‐obtained EV‐Br_2_ aqueous solution (in a molar ration of 2:1) through anion exchange reaction.^[^ ^50^ ^]^ The white precipitation was formed and collected by filtration, washed with deionized (DI) water and freeze‐dried.

### Synthesis of EV‐AQ_2_

The synthesis procedure of EV‐AQ_2_ was similar to that of EV(TFSI)_2_ and synthesized using anionic exchange reaction from EV‐Br_2_ and sodium anthraquinone‐2‐sulfonate (Na‐AQ) in a molar ratio of 1:2. In brief, to a solution of Na‐AQ in DI water (70 mL) was added as‐obtained EV‐Br_2_ powder. Upon the addition, the mixture solution was stirred at 80 °C for 5 h. Then the yellowish solution was allowed to cool to room temperature, and then kept at room temperature for another 3 h. The dark yellow solid thus was filtered, washed with excess DI water, and dried under vacuum at 80 °C for 48 h.

### Cathode Fabrication

A facile dissolution‐recrystallization method was utilized to render a free‐standing cathode for Li‐organic batteries. In detail, commercial binder‐free multiwalled carbon nanotube paper called buckypaper (BP) was used as the electrode discs. BP were punched into Φ12 mm disks and dried at 110 °C for 24 h in a vacuum oven before use. The EV‐AQ_2_ (20 mg) was added to the mixture solvent of DI water and ethanol (400 µL, volume ratio of 3:7). Then, the mixture was heated at 55 °C to form a dissolved solution. Subsequently, the as‐prepared solution (20 µL) was dropped into a piece of the above BP current collector onto a positive plate of CR2032 coin cells and heated at 80 °C for 24 h under vacuum to remove any solvent. The resulting BP electrode was easily adhered to the positive electrode and directly utilized as the free‐standing cathode. The active materials loading (affording a mass of ∼1.0 mg) was calculated by weight of cathode mass. For Na‐AQ electrode, Na‐AQ (20 mg) was dissolved in the mixture solvent of DI water and ethanol (400 µL, volume ratio of 3:7) and heated at 60 °C following the same procedure as mentioned above. To prepare EV‐Br_2_ electrode, the EV‐Br_2_ (20 mg) solution was prepared by dissolving EV‐Br_2_ in methanol to form transparent solution and followed the same procedure above. The binder‐free Na‐AQ/EV‐Br_2_ mixture electrode was constructed by dropping Na‐AQ and EV‐Br_2_ solution into BP discs in turn by controlling the volume of two composites (the molar ratio of Na‐AQ and EV‐Br_2_ was about 2:1). Each material loading was also obtained by weight of cathode mass in order.

### Electrochemical Measurement

The assembling procedure of electrochemical cells was carried out in an Argon‐filled glove box by employing the as‐obtained cathodes. Celgard 2400 was used as the separator. 2 m LiTFSI in G4 served as the electrolyte. Lithium foil was used as the anode for the Li cells. The galvanostatic charge–discharge cycling tests of the resulting CR2032 coin cells were performed on a LAND CT2001A battery tester at 25 °C from 1.7 to 3.0 V at different current densities. The specific capacities were determined based on the weight of the active material in the electrode. Cyclic voltammograms (CV) and electrochemical impedance spectroscopy measurement were performed on a BioLogic VMP‐3 potentiostat. The CV potential was conducted using cutoff voltages of 1.7 and 3.0 V versus Li/Li^+^ at a scanning rate of 0.1 mV s^−1^.

### Chemical Characterization

The XRD data of EV‐Br_2_, Na‐AQ, and EV‐AQ_2_ composites were collected on a Rigaku MiniFlex600 XRD Instrument equipped with Cu *K*_*α*_ radiation. The scanning rate was 2° min^−1^, and 2*θ* was set between 10° and 80°. The Raman measurements were carried out through Xplora with the incident laser of 532 nm in 500–1800 cm^−1^. The morphology and microstructure of these materials were investigated by a Zeiss Sigma 500 SEM apparatus. The elemental mapping was performed with energy‐dispersive X‐ray spectroscopy (EDS) attached to the SEM. A thermogravimetric analyzer was used to determine the thermal stability of composites on STA 409 PC Simultaneous Thermal Analyzer with a temperature range between 30 and 900 °C at 10 °C min^−1^ rate, under argon condition. FTIR absorption spectra were collected to on NEXUS 470 FTIR spectrometer. The scanning region is from 100 to 4000 cm^−1^. NMR spectroscopy was carried out on a Bruker Avance III 400 MHz NMR spectrometer.

## Conflict of Interest

The authors declare no conflict of interest.

## Supporting information

Supporting Information

## Data Availability

The data that support the findings of this study are available from the corresponding author upon reasonable request.
