# Supplementary material for: Biredox‐Ionic Anthraquinone‐Coupled Ethylviologen Composite Enables Reversible Multielectron Redox Chemistry for Li‐Organic Batteries
Source: Adv Sci (Weinh). 2021 Oct 29;9(1):2103632. doi: 10.1002/advs.202103632 (PMC8728824; doi:10.1002/advs.202103632)
Supplement: Supplementary file 1 — Supporting Information [file ADVS-9-2103632-s001.pdf]

WILEY-VCH

© 2021 Wiley-VCH GmbH

ADVANCED SCIENCE
Open Access

# Supporting Information

for *Adv. Sci.*, DOI: 10.1002/advs.202103632

# Bi-redox-ionic Anthraquinone-coupled Ethylviologen Composite Enables Reversible Multi-electron Redox Chemistry for Li-organic Batteries

*Zhongju Wang, Qianqian Fan, Wei Guo, Changchun Yang, and Yongzhu Fu**

Supporting Information

**Bi-redox-ionic Anthraquinone-coupled Ethylviologen Composite Enables Reversible Multi-electron Redox Chemistry for Li-organic Batteries**

*Zhongju Wang, Qianqian Fan, Wei Guo, Changchun Yang, and Yongzhu Fu**

**Supporting figures:**

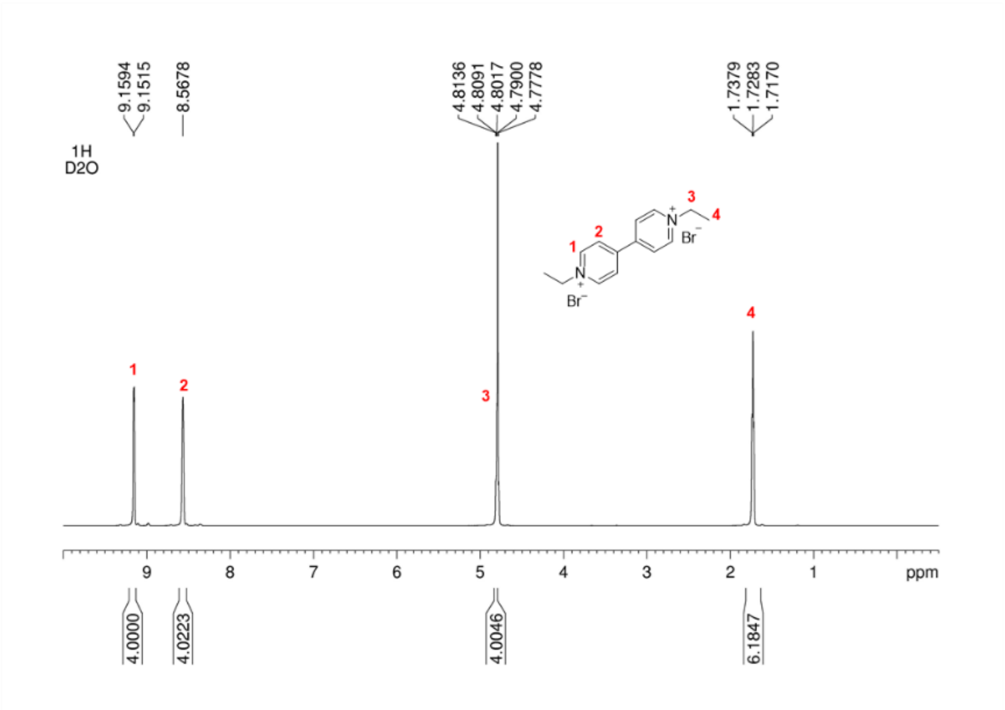


**Figure S1.** $^1$H liquid NMR spectrum of the as-prepared EV-$Br_2$ measured in $D_2O$.

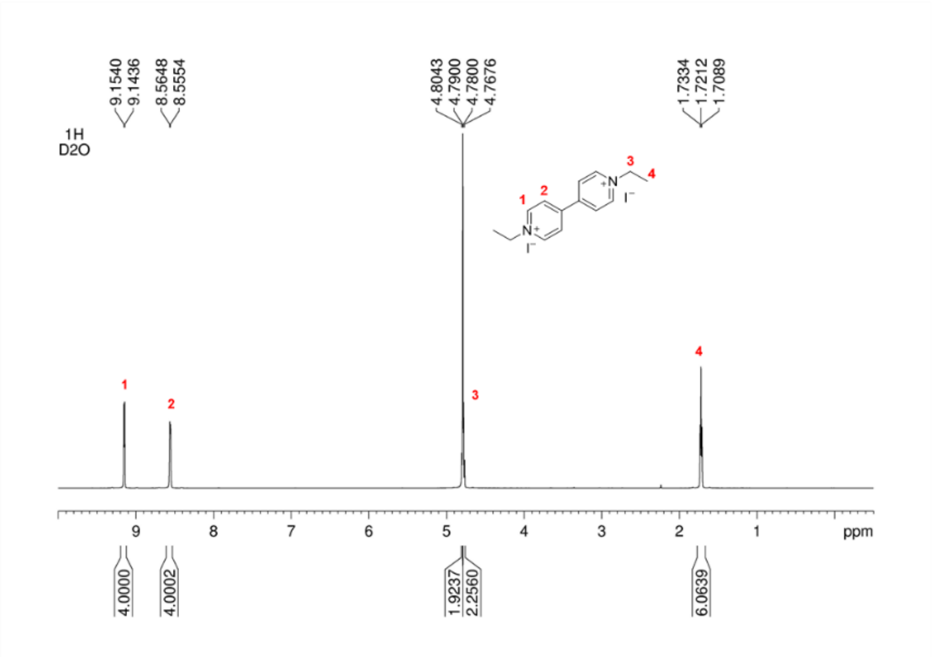


**Figure S2**. $^{1}$H liquid NMR spectrum of the as-prepared EV-$I_2$ measured in $D_2O$.

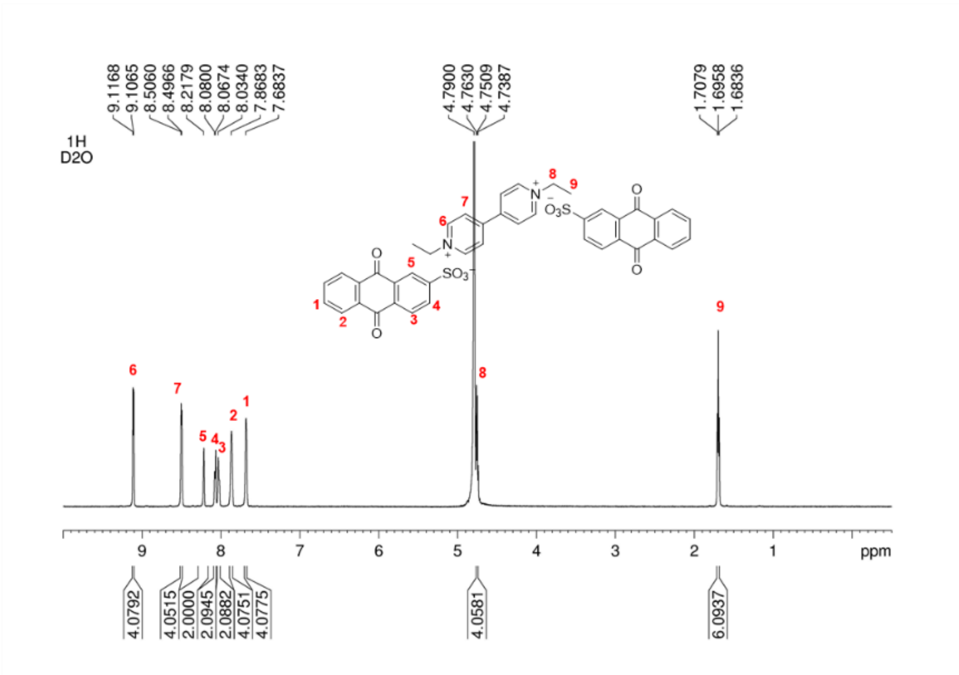


**Figure S3.** $^{1}$H liquid NMR spectrum of the as-prepared EV-$AQ_2$ measured in $D_2O$.

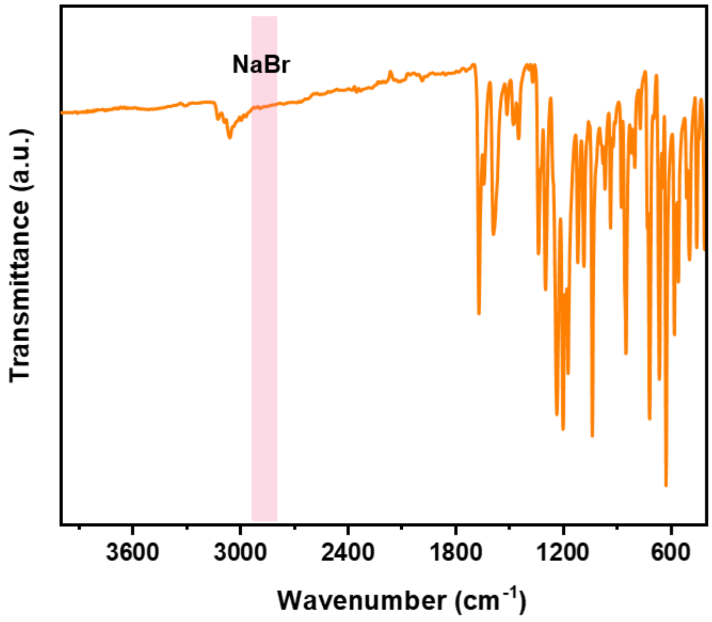


**Figure S4.** Full FTIR spectrum of EV-$AQ_2$.

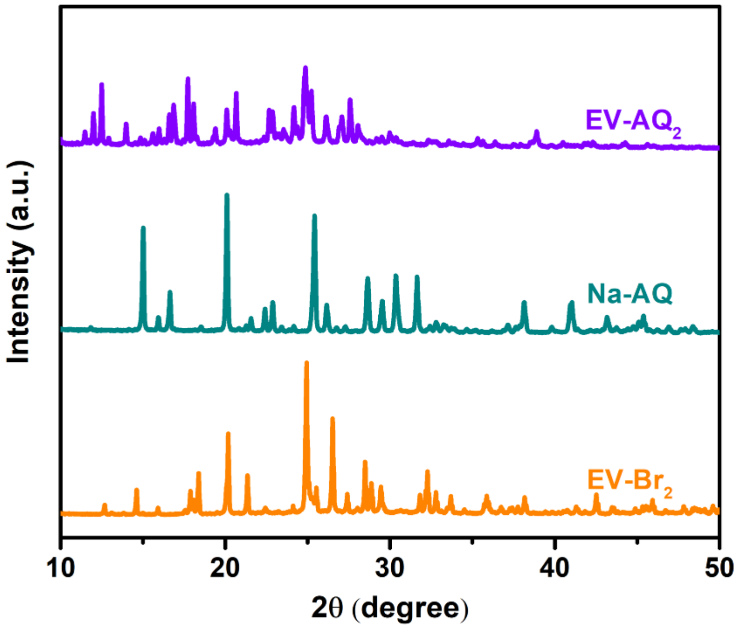


**Figure S5.** XRD patterns of EV-$Br_2$, Na-AQ, and EV-$AQ_2$.

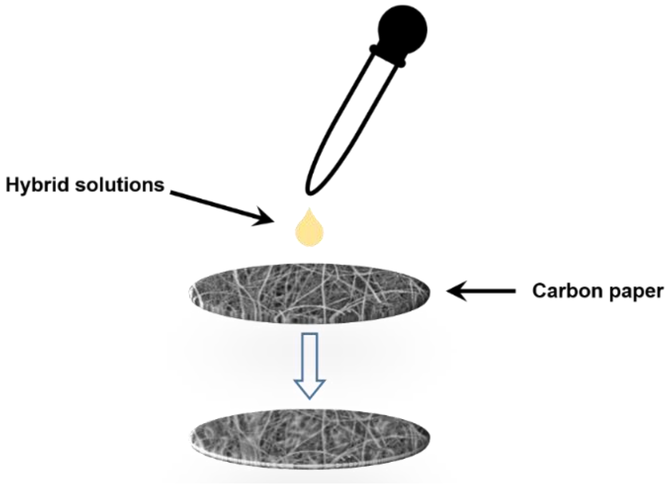


**Figure S6.** Schematic of the electrode configuration through a facile dissolution-recrystallization method.

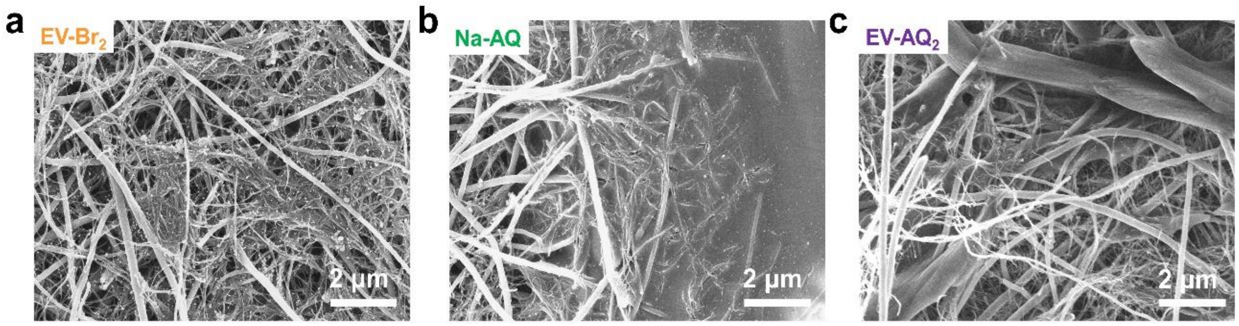


**Figure S7.** SEM images of (a) EV-$Br_2$, (b) Na-AQ, and (c) EV-$AQ_2$ cathode.

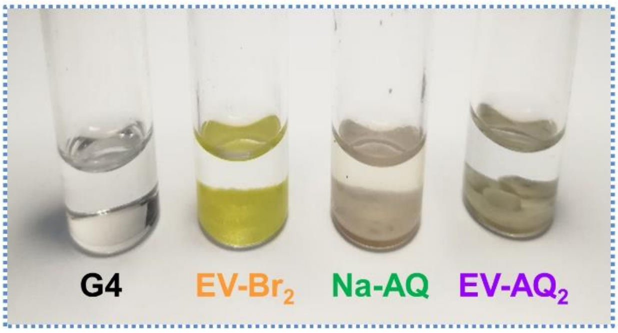


**Figure S8.** Digital photos of G4 solutions before and after the addition of $EV\text{-}Br_2$, Na-AQ, and $EV\text{-}AQ_2$, respectively.

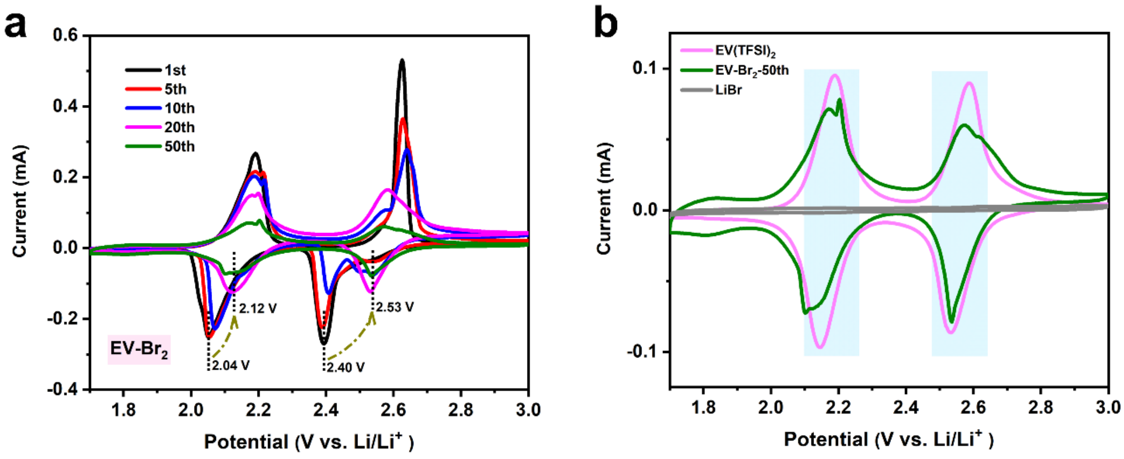


**Figure S9.** (a) Cyclic voltammograms of $EV\text{-}Br_2$ cathode in the 1st, 5th, 10th, 20th, and 50th cycle in the voltage range of 1.7 V-3.0 V. (b) Cyclic voltammograms of LiBr cathode, $EV(TFSI)_2$ cathode, and $EV\text{-}Br_2$ cathode in 50th cycle in the voltage range of 1.7 V-3.0 V. All scan rates were kept at a scan rate of 0.1 mV $s^{-1}$.

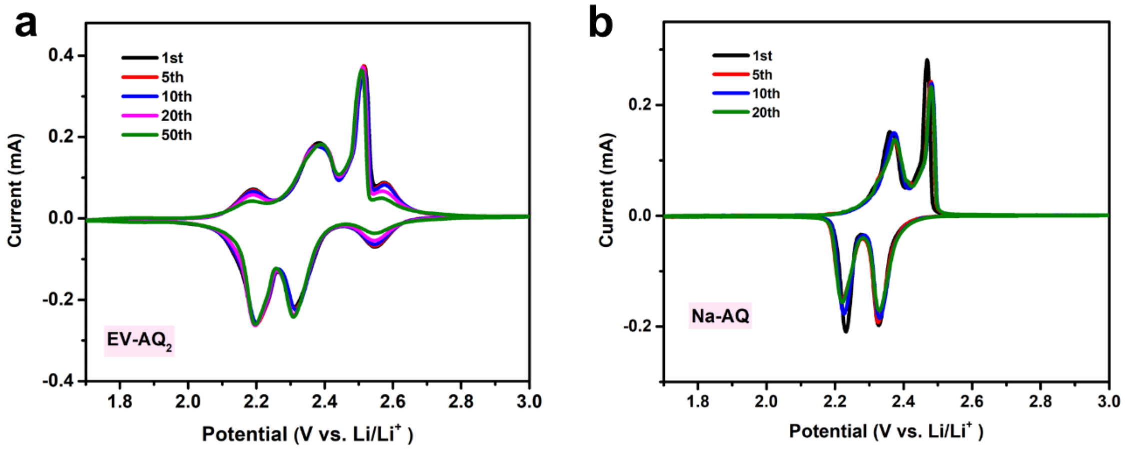


**Figure S10.** (a) Cyclic voltammograms of EV-$AQ_2$ cathode in the 1st, 5th, 10th, 20th, and 50th cycle. (b) Na-AQ cathode in the 1th, 5th, 10th and 20th cycle in the voltage range of 1.7 V-3.0 V at a scan rate of 0.1 mV $s^{-1}$.

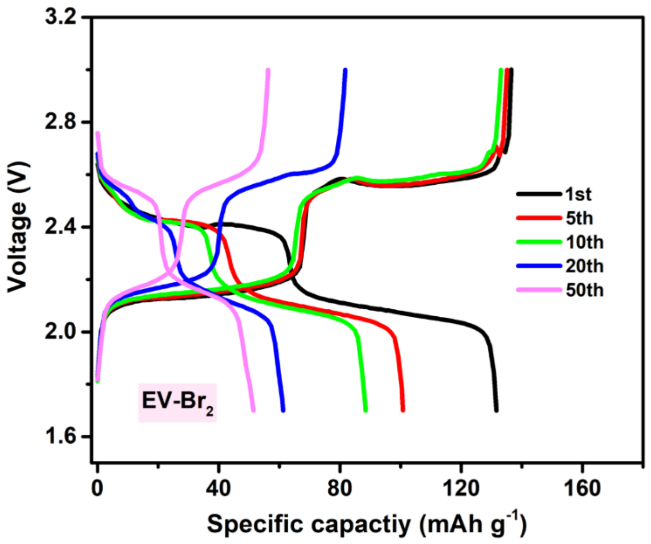


**Figure S11.** Galvanostatic charge/discharge profiles of EV-$Br_2$ cathode during different cycles at 0.1 C (1C = 143.3 mA $g^{-1}$).

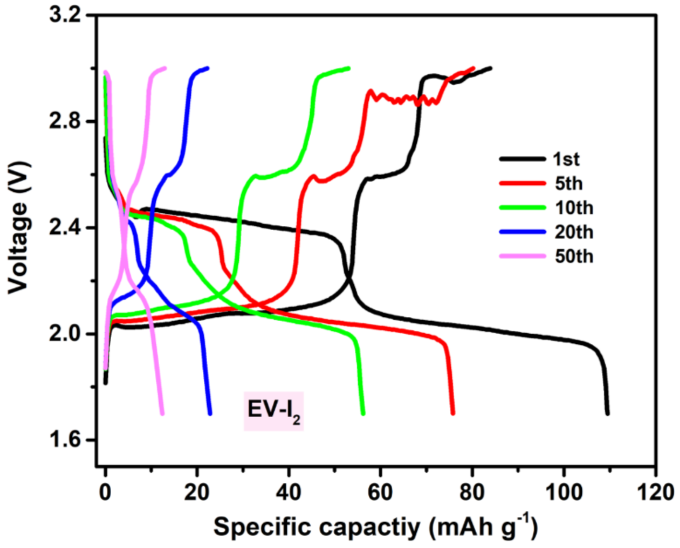


**Figure S12.** Galvanostatic charge/discharge profiles of EV-$I_2$ cathode of different cycles at 0.1 C rate (1C = 114.5 mA $g^{-1}$).

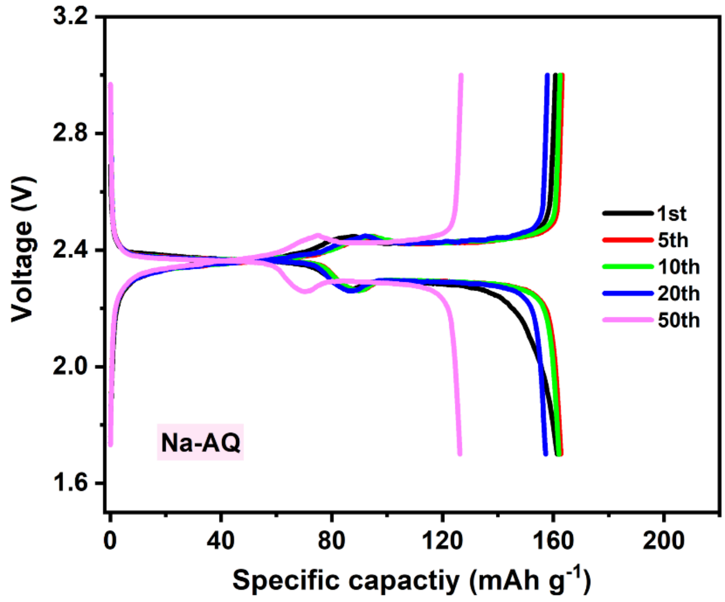


**Figure S13.** Galvanostatic charge/discharge profiles of Na-AQ cathode during different cycles at 0.1 C (1 C = 172.8 mA $g^{-1}$).

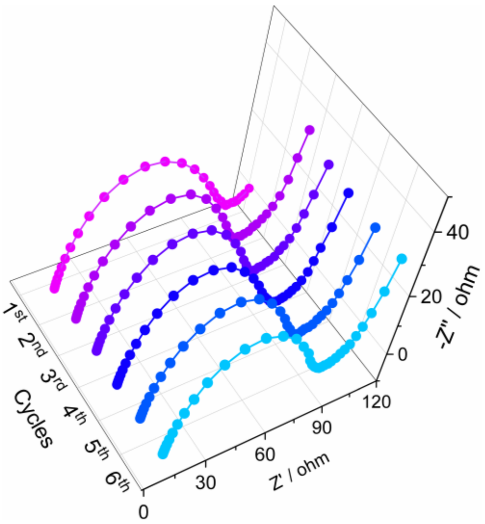


**Figure S14.** *In-situ* electrochemical impedance spectra of EV-$AQ_2$ cathode during cycling.

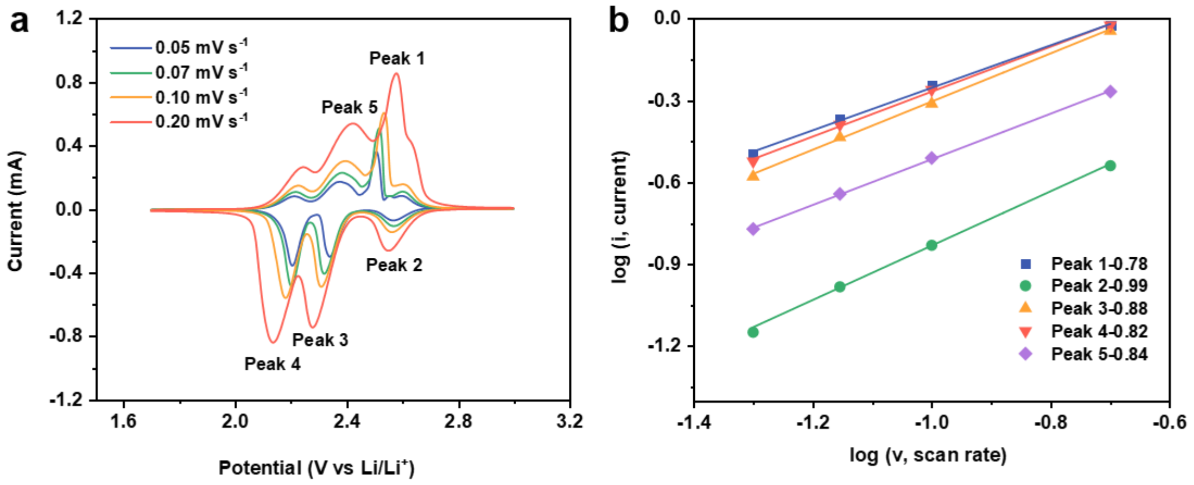


**Figure S15.** Kinetic property investigations of the redox reaction on EV-$AQ_2$ cathode. (a) CV curves of EV-$AQ_2$ electrode at scan rates ranging from 0.05 mV $s^{-1}$ to 0.2 mV $s^{-1}$ in the voltage range of 1.7-3.0 V vs. $Li/Li^+$. (b) Log(i)~log(v) plots of anodic and cathodic peaks derived from the CV curves, the corresponding b values are 0.78, 0.99, 0.88, 0.82, and 0.84, respectively.The achieved voltammetry responses at different scan rates are summarized as $i = av^b$, where i is peak current (mA), v is scan rate, and a, b are constant values.[1] The calculated b value is higher than 0.78 for both the anodic peaks and cathodic peaks, meaning the fast kinetic and pseudocapacitive behavior (b>0.5) without the diffusion control.

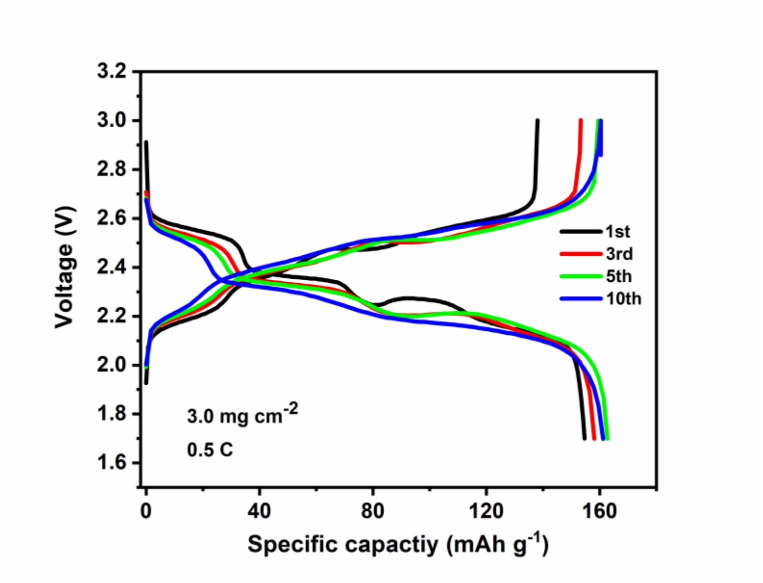


**Figure S16.** Galvanostatic charge/discharge curves of EV-$AQ_2$ cathode with high areal loading of 3 mg cm$^{-2}$ at 0.5 C.

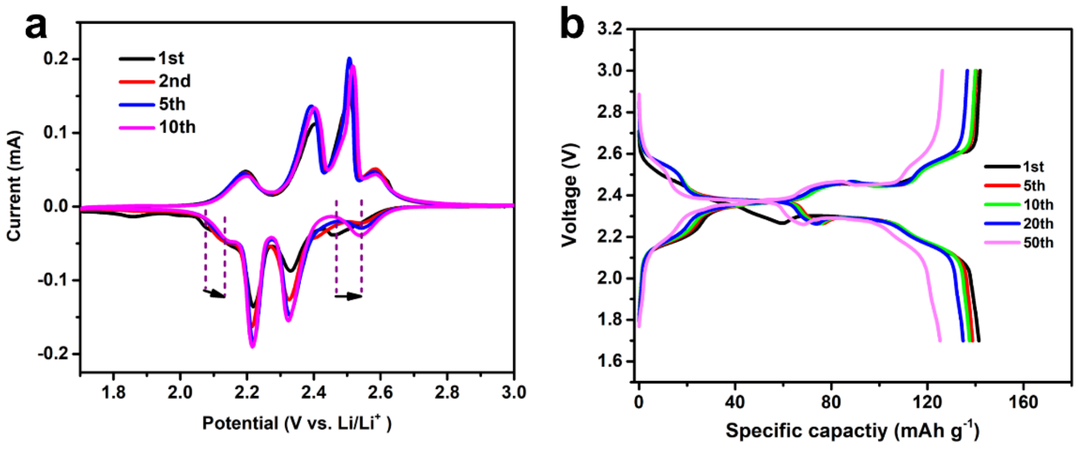


**Figure S17.** (a) CV curves of the mixture cathode of EV-$Br_2$ and Na-AQ in the 1st, 2nd, 5th, and 10th cycle at a scan rate of 0.1 mV s$^{-1}$ in the voltage range of 1.7 V-3.0 V. (b) Galvanostatic charge/discharge curves of the mixture cathode of EV-$Br_2$ and Na-AQ at a current rate of 0.2 C within the voltage range of 1.7 V-3.0 V.

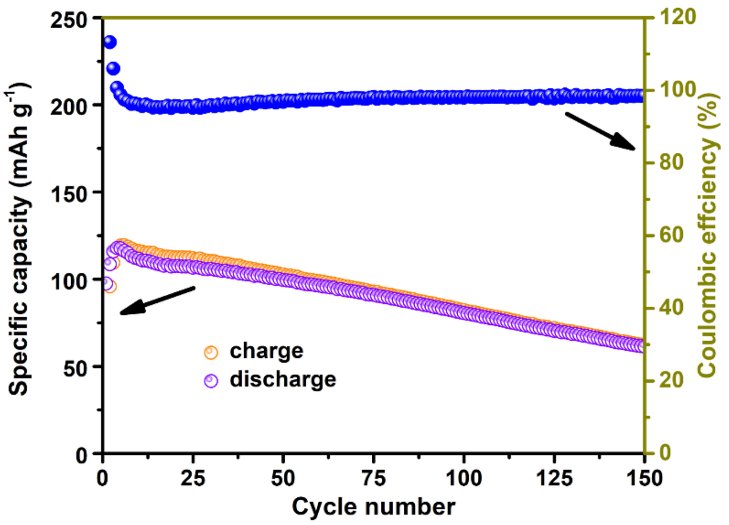


**Figure S18.** Cycling performance of the mixture cathode of EV-$Br_2$ and Na-AQ at a current rate of 0.2 C within the voltage range of 1.7 V-3.0 V.

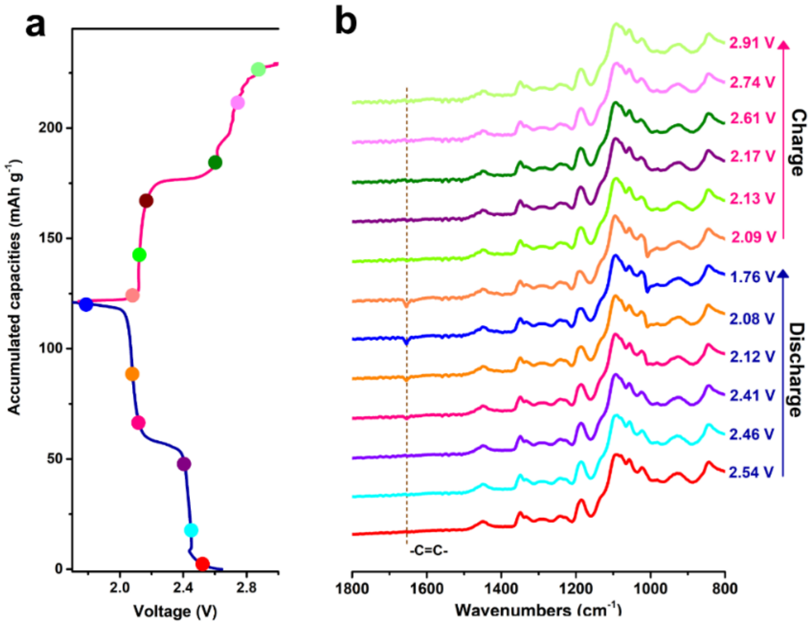


**Figure S19.** (a) Galvanostatic charge/discharge profiles of EV-$Br_2$ cathode at 0.1 C with marked points at different discharge and charge states in the *in-situ* FTIR tests. (b) *In-situ* FTIR spectra of EV-$Br_2$ cathode taken at different states as marked in (a).

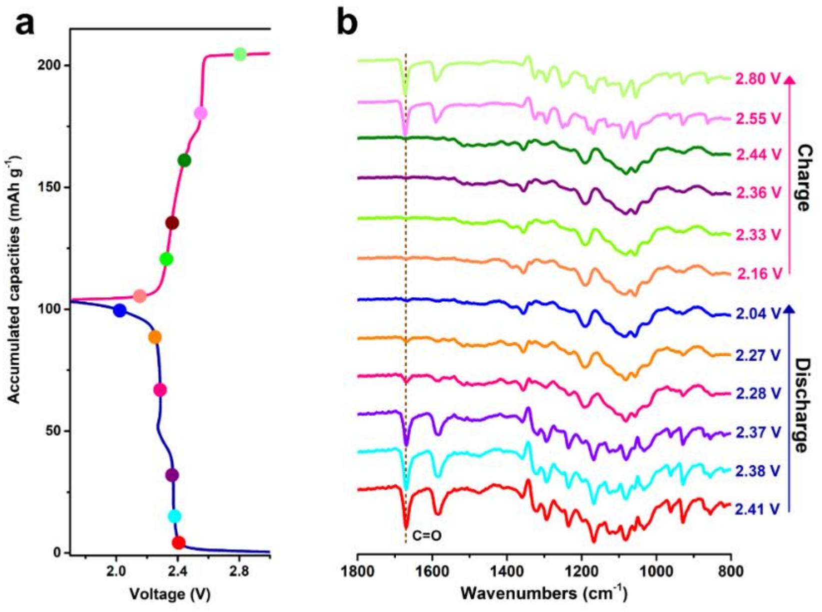


**Figure S20.** (a) Galvanostatic charge/discharge profiles of Na-AQ cathode at 0.1 C with marked points at different discharge and charge states in the *in-situ* FTIR tests. (b) *In-situ* FTIR spectra of Na-AQ cathode taken at different states as marked in (a).

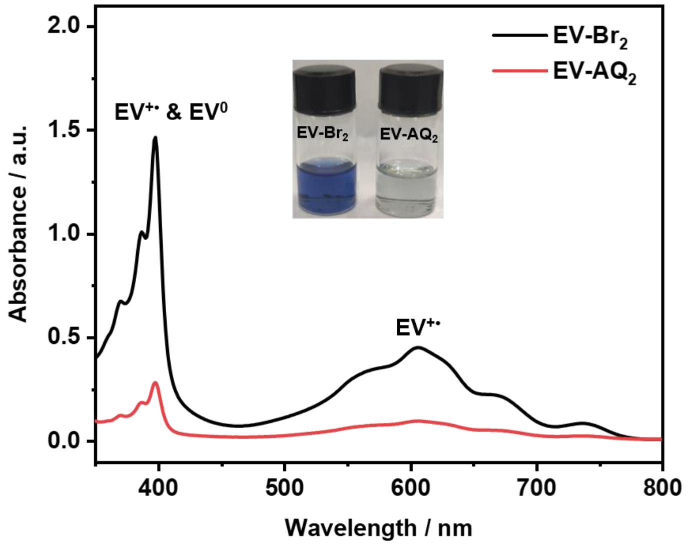


**Figure S21.** UV-vis spectra of the recharged EV-$AQ_2$ and EV-$Br_2$ cathodes after several cycles.

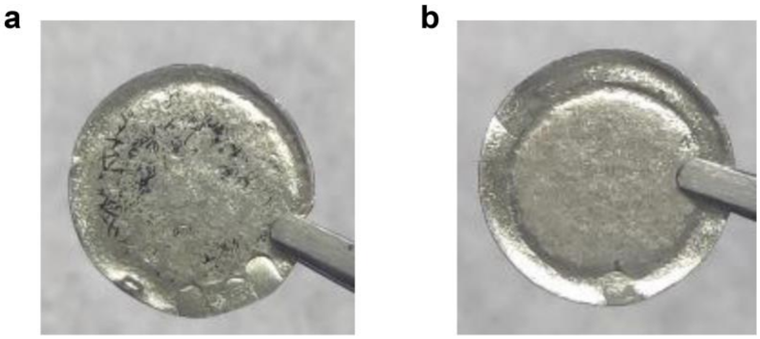


**Figure S22.** SEM imgaes of lithium anodes paried with (a) EV-$Br_2$ and (b) EV-$AQ_2$ cathodes after several cycles.

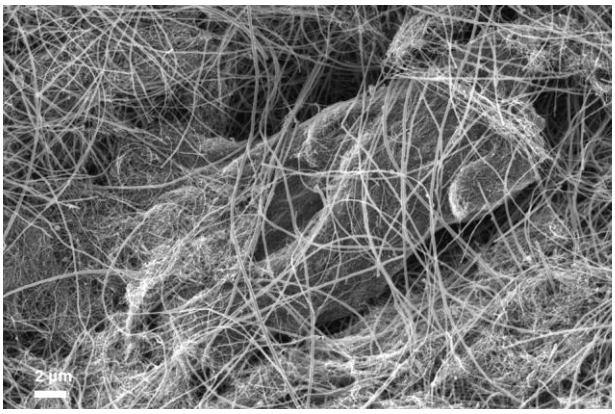


**Figure S23.** SEM image of the recharged EV-$AQ_2$ cathode after several cycles in battery.

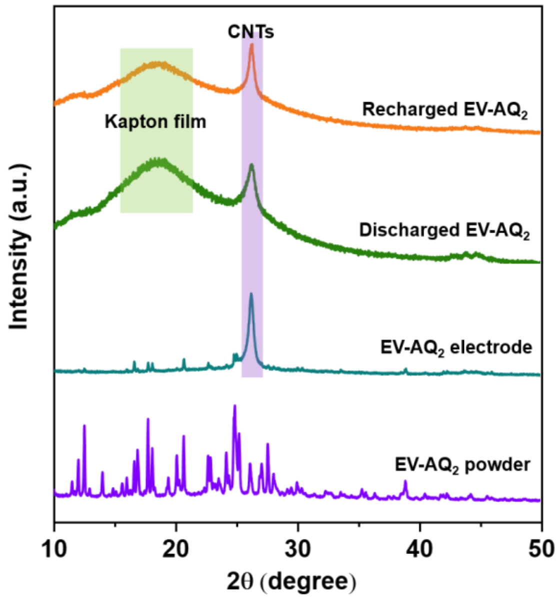


**Figure S24.** *Ex-situ* XRD pattern of the discharged and recharged EV-$AQ_2$ cathodes after several cycles.

**Supplementary references**

[1] P. Simon, Y. Gogotsi, B. Dunn, *Science* 2014, **343**, 1210-1211.
